# Inhibition of mTOR downregulates expression of DNA repair proteins and is highly efficient against BRCA2-mutated breast cancer in combination to PARP inhibition

**DOI:** 10.18632/oncotarget.25640

**Published:** 2018-07-03

**Authors:** Rania El Botty, Florence Coussy, Rana Hatem, Franck Assayag, Sophie Chateau-Joubert, Jean-Luc Servely, Sophie Leboucher, Charles Fouillade, Sophie Vacher, Bérengère Ouine, Aurélie Cartier, Leanne de Koning, Paul Cottu, Ivan Bièche, Elisabetta Marangoni

**Affiliations:** ^1^ Institut Curie, PSL Research University, Translational Research Department, 75005 Paris, France; ^2^ Genetics Department, Institut Curie, PSL Research University, 75005 Paris, France; ^3^ Medical Oncology Department, Institut Curie, PSL Research University, 75005 Paris, France; ^4^ BioPôle Alfort, Ecole Nationale Vétérinaire d’Alfort, 94700 Maisons Alforts, France; ^5^ INRA, PHASE Department, UMR3306, 75338 Paris, France; ^6^ Institut Curie, PSL Research University, UMR3306, 91405 Orsay, France; ^7^ Institut Curie, PSL Research University, Inserm U 612, Centre Universitaire, 91405 Orsay, France

**Keywords:** BRCA2, breast cancer, mTOR, DNA repair, PDX

## Abstract

Breast cancer is a complex disease in which each patient could present several genetic alterations that are therapeutically relevant in cancers. Here we explored the therapeutic benefit of combining PARP and mTOR inhibitors in a context of DNA repair deficiency and PI3K pathway activation.

The combination of everolimus and olaparib was tested in BRCA2-mutated patient-derived xenografts (PDX) carrying alterations in the PI3K/AKT/mTOR pathway. An RPPA analysis of different signalling pathways was performed in untreated and treated xenografts.

Everolimus and olaparib showed marked anti-tumor activities in the monotherapy setting and high efficacy when given in combination with 100% of mice showing tumor regressions. The fraction of P-H2AX positive cells was increased in both monotherapy arms and strongly increased in the combination setting. Everolimus given as monotherapy resulted in downregulation of different proteins involved in DNA damage repair, including FANCD2, RAD50 and SUV39H1. In the combination setting, expression of these proteins was almost completely abolished, suggesting convergence of PARP and mTOR in downregulation of DNA damage repair components.

In conclusion, our results suggest that combining mTOR and DNA repair inhibition could be a successful strategy to treat a subset of breast cancer with BRCA2 mutation and alterations in the PI3K/AKT/mTOR pathway.

## INTRODUCTION

Current treatment options for breast cancer are moving toward potent targeted therapies in general well tolerated and that can be tailored to an individual patient’s tumor. There are now targeted therapeutic options available for nearly all breast cancer subtypes, exploiting the differing drivers of carcinogenesis within these individual tumors [1]. Indeed, a better understanding of the biology of breast cancer and the recent advances in the application of genomic technologies led to the identification of a number of molecular targets. Breast cancer is as a complex disease in which each tumor presents several genomic alterations and activated pathways [2, 3]. Therefore, there is a strong rationale to combine drugs based on the presence of multiple genomic alterations and/or pathway activation [4]. Among them are tyrosine kinase inhibitors directed at a number of targets (HER1, HER2, HER3, IGF receptor [IGFR], C-MET, FGF receptor [FGFR]), inhibitors of intracellular signaling pathways (PI3K, AKT, mammalian target of rapamycin [mTOR]), angiogenesis inhibitors and agents that interfere with DNA repair [5]. Some of these agents have shown remarkable activity and have become part of the standard of care in patients with breast cancer (exemplified by the anti-HER2 agents trastuzumab and lapatinib). Others have been recently approved for the treatment of specific breast cancer subtypes, such as the mTOR inhibitor everolimus in advanced luminal breast cancer and the poly(ADP-ribose)polymerase (PARP) inhibitor olaparib in metastatic breast cancer with germline BRCA1 or BRCA2 mutations [6–9].

One of the challenges that physicians are confronted with, is the ability to match each patient with the right therapy. Given the complexity of the cancer cell signal transduction networks, it may be more rational to inhibit more than one target or pathway at a time. Choices on drug combinations in clinical studies should be based on biological rationales and preclinical evidence of additive or synergistic effects.

DNA repair deficiencies and activation of PI3K pathway are relatively common events in breast cancer. BRCA1/2 mutations have been associated with sensitivity to PARP1 inhibitors (synthetic lethality) and DNA alkylating agents (genotoxic) [9], while alterations in component of the PI3K pathway might confer sensitivity to PI3K and mTOR inhibitors.

The mTOR inhibitor everolimus has been recently approved for the treatment of advanced ER+ breast cancer in a context of endocrine resistance [6]. Although everolimus combined with an aromatase inhibitor improved progression-free survival, not all patients respond to everolimus and even those who respond eventually develop resistance [10]. Patient stratification and predictive biomarker are still lacking [11].

Here we explored the therapeutic benefit of combining everolimus to a PARP inhibitor in two different patient-derived xenografts of BRCA2-mutated breast cancer with genomic alterations in the PI3K pathway.

We show that everolimus combined to olaparib lead to unrepaired DNA damage and tumor regression *in vivo*, through a cross-talk between DNA repair and mTOR pathways.

## RESULTS

### Treatment of a BRCA2 mutated luminal breast cancer PDX by everolimus and olaparib results in tumor regression

To test whether the combination of DNA repair and mTOR inhibitors could be a relevant therapeutic strategy in tumors with genomic alterations in DNA repair and PI3K/AKT/mTOR pathway, we treat a PDX model established from a BRCA2 mutated breast cancer (HBCx-22 TamR) with the Parp inhibitor olaparib combined to the mTOR inhibitor everolimus.

This PDX model has been established from an ER+ primary breast cancer [12] and has been rendered resistant to tamoxifen in mice, through long-term *in vivo* treatment and re-engraftment of xenograft that showed acquired resistance to tamoxifen, as previously described [13]. This tumor carries an in frame deletion in the *PIK3R1* gene and a frameshift inactivating mutation of *PTEN* associated to its protein loss (Table 1).

**Table 1:** Breast cancer subtypes and genomic characteristics of BRCA2-mutated PDX models

	HBCx22 TamR	HBCx-17
Subtype	Luminal B	Basal-like
Germline BRCA2 mutation	c.6405_6409del5, p.Asn2135Lysfs^*^3 (NM_000059.3)	c.6033_6034del, p.Ser2012GlnFs^*^5 (NM_000059.3)
*PIK3R1*	c.1704_1727del, p.Arg569_Thr576del (NM_181523.1)	Wild-type
*PI3KCA*	Wild-type	Wild-type
*PTEN*	c.314G>T, p.Cys105Phe (NM_000314.4) LOH and protein loss	Intragenic deletion LOH and protein loss
Reference	Cottu et al. (2014)	De Plater et al. (2010)

**Figure 1:**
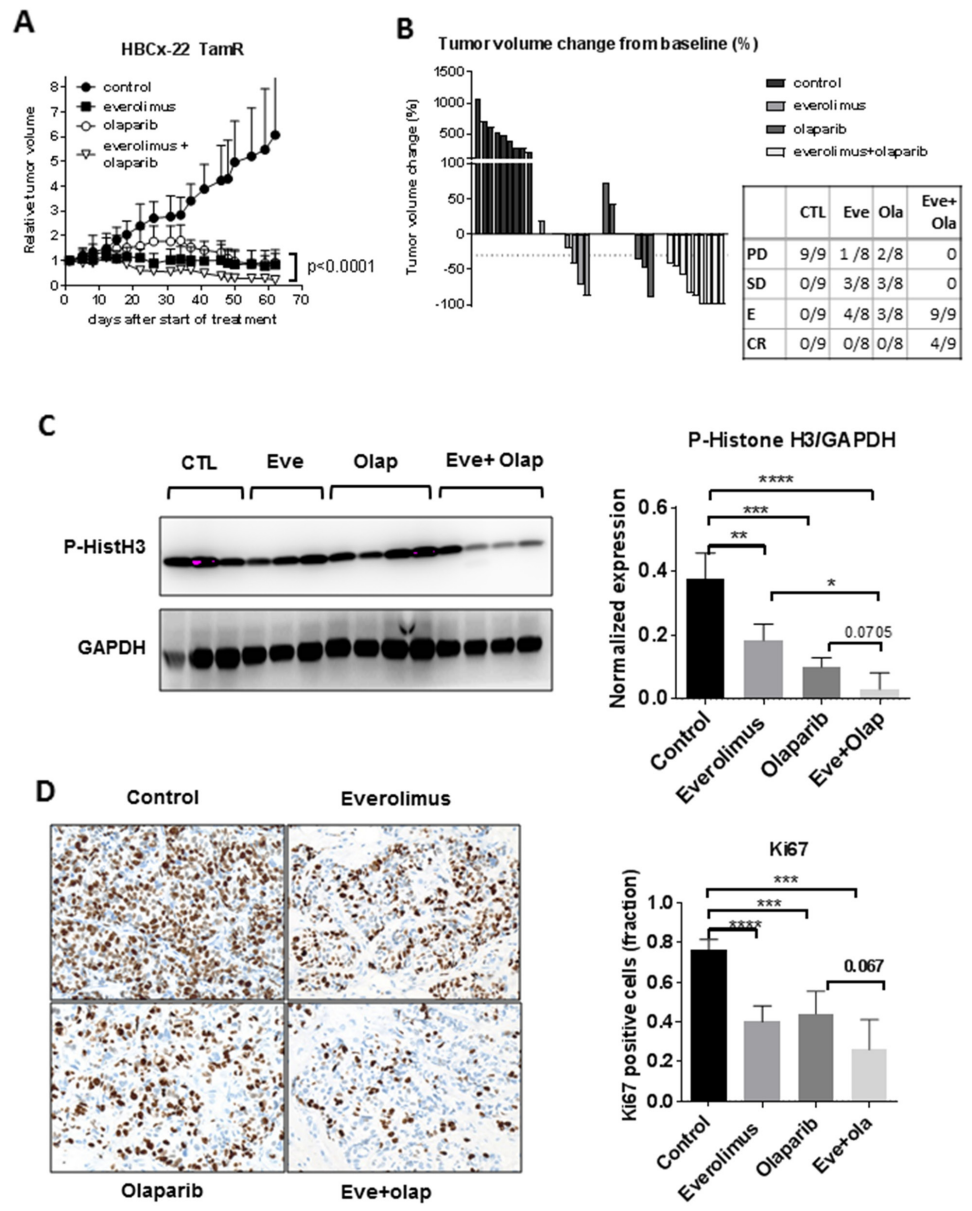
Anti-tumour activity of everolimus and olaparib in a BRCA2-mutated ER+ breast cancer **(A)** tumour growth curves of the HBCx-22 TamR xenograft treated by olaparib, everolimus and the combination. **(B)** waterfall plot displayed as percent of tumour volume change from baseline (each bar is an individual xenograft). **(C)** Western blot analysis of Histone H3 phosphorylation in HBCx22 TamR. **(D)** IHC analysis of Ki67 in the HBCx-22 TamR xenografts. Quantitative analysis of Ki67-positive cells was performed on technical replicates (N=5) and counting >100 nuclei. Statistical analysis of normalized P-histone H3/GAPDH and Ki67 staining between groups was performed by the unpaired *t*-test.^*^p<0.05; ^**^p<0.005; ^***^p< 0.0005.

The effect of everolimus, olaparib and evero-limus+olaparib treatments on the tumor growth of HBCx-22 TamR are shown in Figure 1A and 1B. In the monotherapy setting, everolimus and olaparib inhibited tumor growth with a TGI of 86% with 4 and 3 mice showing tumor regression, respectively (Figure 1B). The combination of the two treatments resulted in strong tumor growth inhibition (TGI of 96%) with 9/9 mice in tumor regression and 4 complete responses. Tumor growth inhibition was higher in the combination group as compared to the monotherapy groups (p<0.0001 when compared to olaparib and p=0.0002 when compared to everolimus, Mann-Whitney *t*-test).

To analyze the effect of treatments on tumor, a second experiment was performed where treatments were started in larger tumors (tumor volume between 150 and 400 mm3), were administered during 3 weeks and samples were harvested before complete response occurred (3 hours after the last treatment). We then analyzed the expression of phospho-histone H3 (P-HistH3) and Ki67 by western blot and immunohistochemistry analysis, respectively. Expression of P-HistH3 and Ki67 were inhibited in both monotherapy groups and strongly reduced in the combination arm (Figure 1C and 1D). In summary, these results show that treatment by everolimus combined to olaparib results in strong tumor growth inhibition with 100% of animals showing tumor regression in a PDX of ER+ BRCA2 mutated breast cancer.

### Treatment by everolimus results in increased DNA damage and decreased expression of several proteins involved in DNA repair

**Figure 2:**
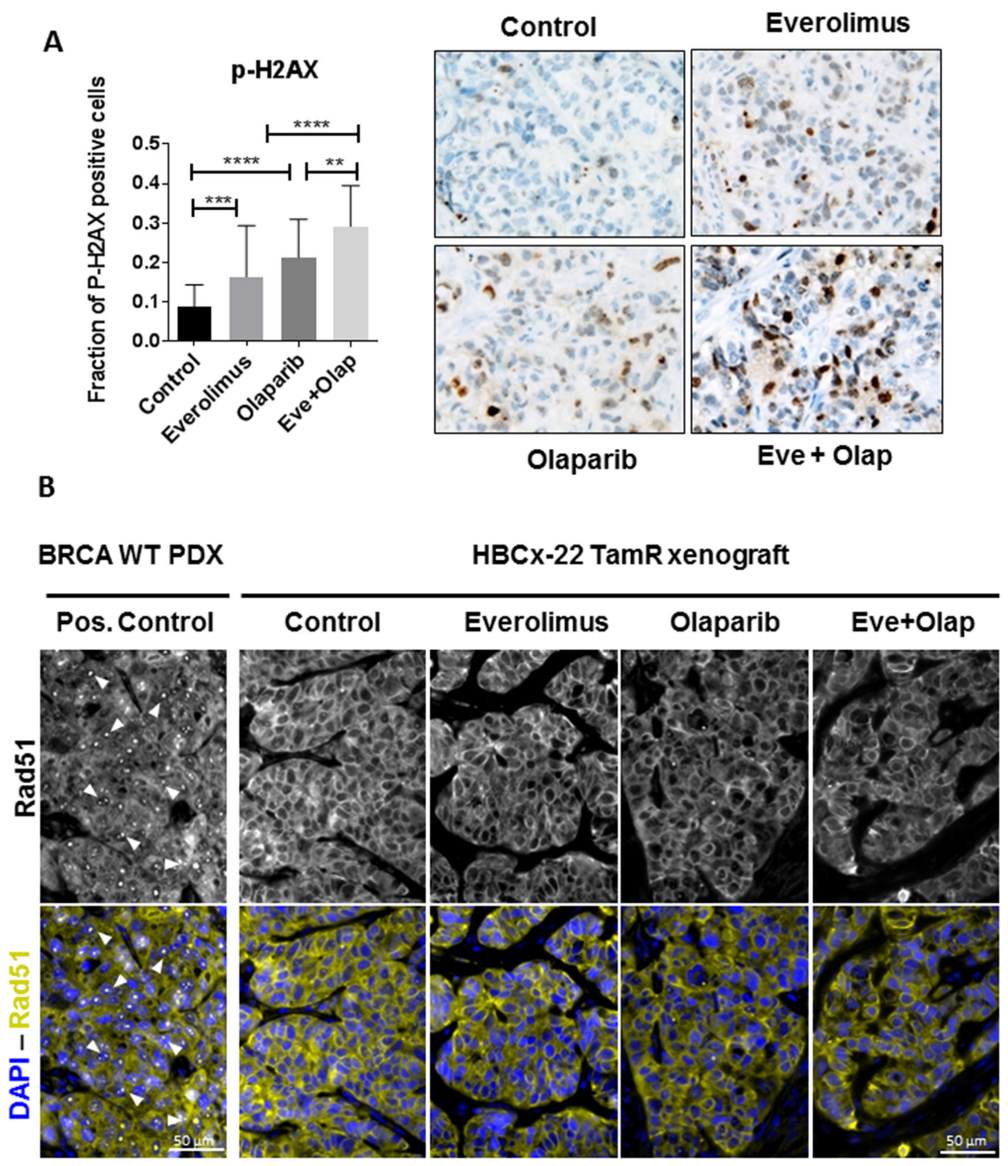
Analysis of DNA damage and expression of DNA repair proteins in HBCx22 TamR **(A)** Fraction of P-H2AX positive cells in HBC-x22-TamR xenografts as determined by IHC analysis. N=5 xenografts/group. Representative images of P-H2AX (40X). **(B)** Representative images (40X) of RAD51 foci in the HBCx-22 TamR xenografts and in BRCA1/2 wild-type PDX as positive control.

The presence of double-strand break (DSB) in chromatin induces the phosphorylation of the histone H2AX, and this modification can be used as a marker for mechanistic studies of DSB induction and repair (44–46). To test the effect of olaparib and everolimus on DSB induction, we measured phosphorylation of H2AX (P-H2AX) by immunohistochemistry in tumors treated during 3 weeks and harvested 3 h after the last dose. Tumors treated with everolimus and olaparib alone showed an increased faction of P-H2AX positive cells (16% and 21% respectively, compared to 8% in the control group), indicating that not only the DNA repair inhibitor olaparib could increase DNA damage (as it is expected) but also the mTOR inhibitor everolimus (Figure 2A). In the combination arm, the fraction of P-H2AX was significantly higher than in both monotherapy groups, with 29% of P-H2AX positive cells. The formation of RAD51 foci in HBCx-22 TamR xenografts was impaired (Figure 2B), confirming that this BRCA2 deficient PDX is unable to repair DNA double strands breaks by homologous recombination.

To identify putative crosstalk events between mTOR and DNA repair, a Reverse Phase Protein Array (RPPA) analysis of multiple signaling pathways and DNA repair processes was performed on untreated and treated xenografts.

The list of antibodies is provided in Supplementary Table 1 and covered different signaling pathways (DNA repair, cell-cyle, PI3K/AKT/mTOR and MAPK pathways, apoptosis and chromatin remodeling pathways). Raw data of RPPA results are showed in Supplementary Table 2.

**Figure 3:**
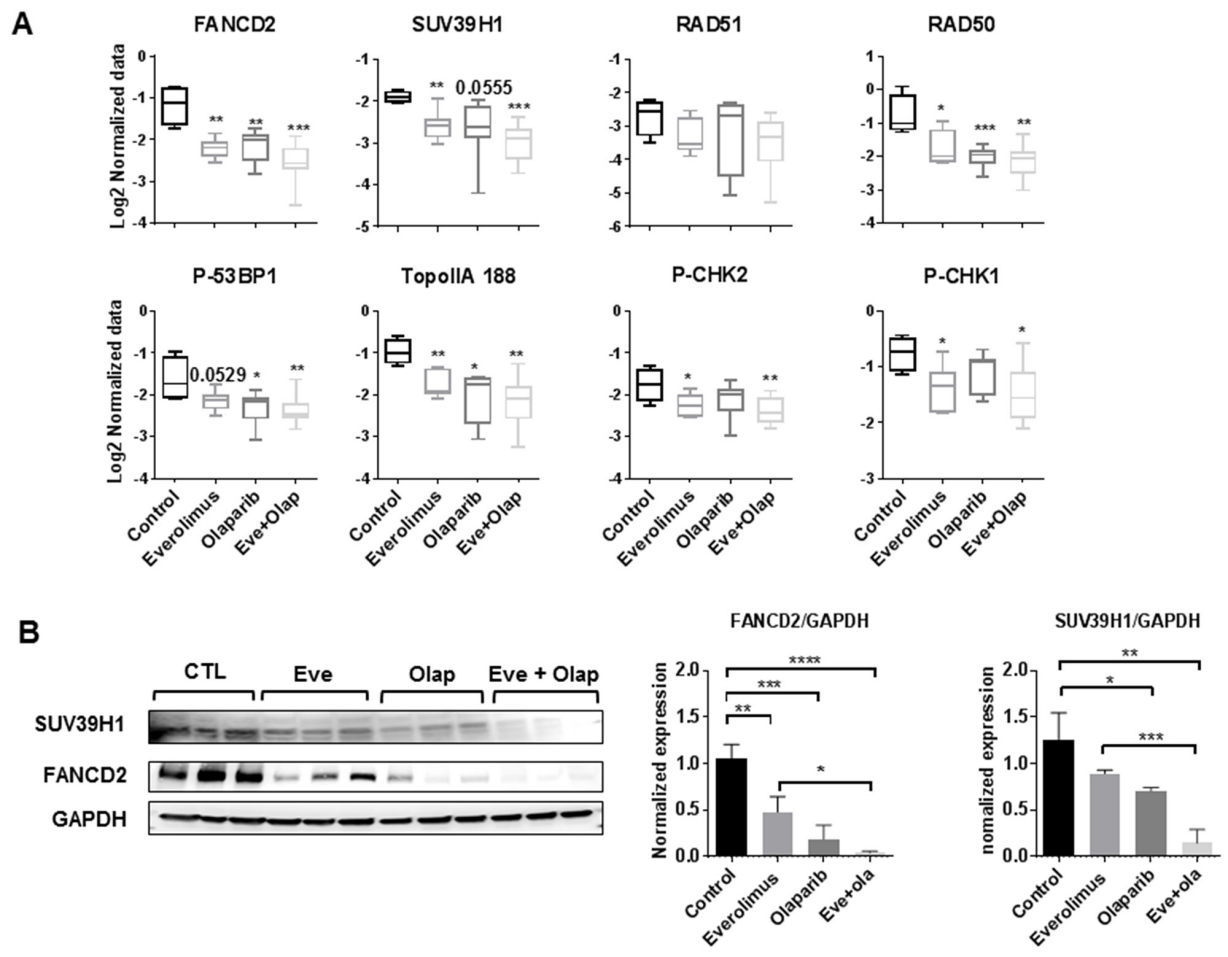
**(A)** RPPA analysis of selected proteins involved in DNA repair, chromatin remodeling and cell cycle. Statistical analysis between groups was performed by the unpaired *t*-test.^*^p<0.05; ^**^p<0.005; ^***^p< 0.0005. **(B)** Western blot validation of SUV39H1 and FANCD2 inhibition in treated xenografts. N=3 xenografts/group

Results of RPPA analysis indicate that treatment by everolimus and olaparib, both in the monotherapy and combination settings, decreased expression of several proteins involved in homologous recombination (RAD50, FANCD2, P-p53BP1), checkpoint control (P-CHK1 and P-CHK2), Toposimerase II alpha and chromatin remodeling (MST1 and SUV39H1) (Figure 3A). FANCD2 protein belongs to the Fanconi Anemia Pathway and is required for repair of DSB and intra-S-phase checkpoint activation [14], while SUV39H1 is a methyltransferase that directs H3K9 methylation on large chromatin domains adjacent to the DSB to promote activation of DSB-signaling proteins [15]. Inhibition of FANCD2 and SUV39H1 protein expression in HBCx22 TamR xenografts was confirmed by Western Blot analysis (Figure 3B).

**Figure 4:**
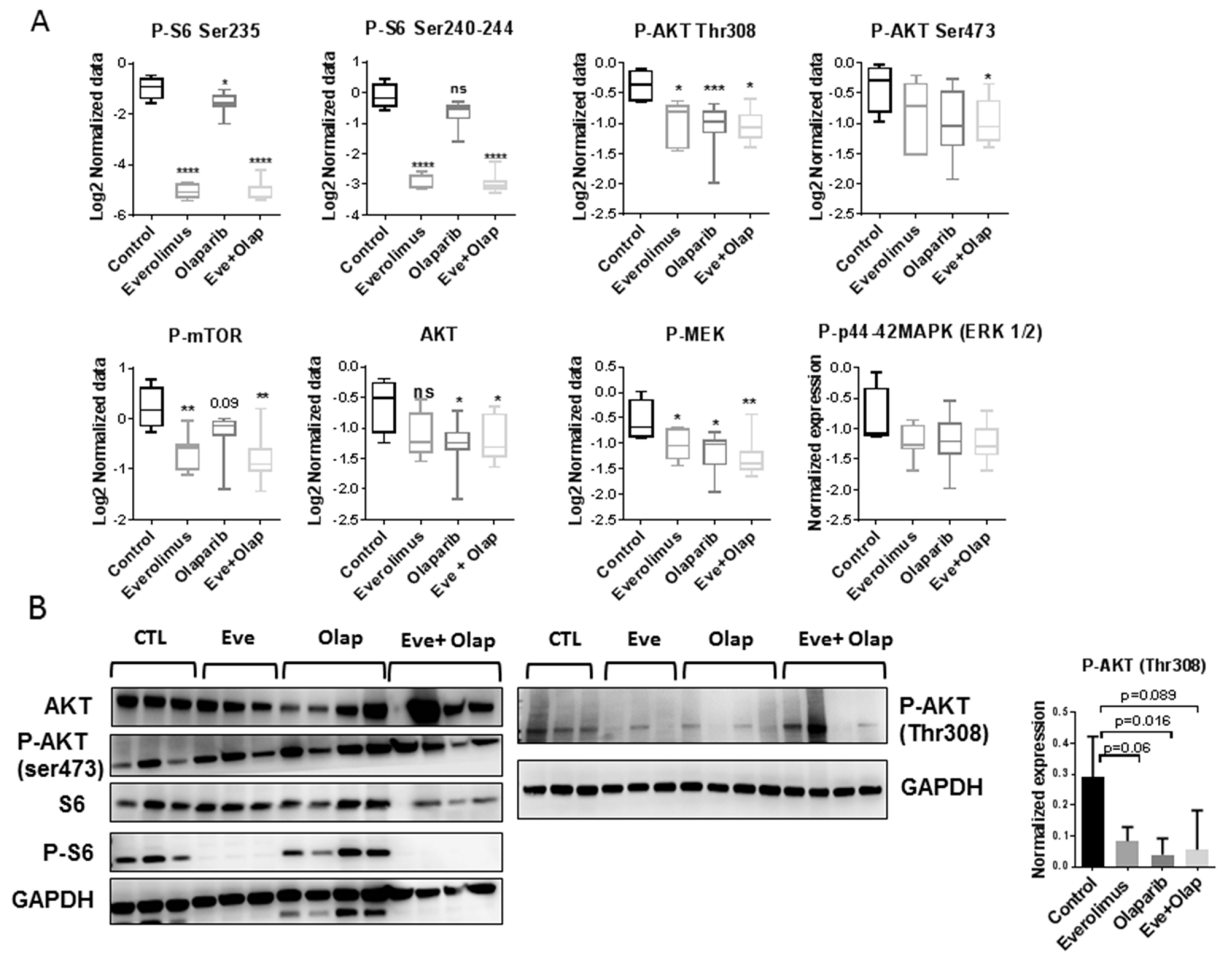
Analysis of PI3K/AKT/mTOR and MAPK pathways **(A)** RPPA analysis of selected proteins involved in PI3K/AKT/mTOR and MAPK pathways. Statistical analysis between groups was performed by the unpaired *t*-test.^*^p<0.05; ^**^p<0.005; ^***^p< 0.0005. **(B)** Western blot validation of P-AKT and P-S6 inhibition in treated xenografts. N=3 or 4 xenografts/group. Normalized expression level of P-AKT (Thr308) determined by western blot analysis.

RPPA analysis of proteins involved in PI3K and MAPK pathways showed strong inhibition of P-S6 in the everolimus treated xenografts (monotherapy and combination groups) and inhibition of AKT phosphorylation (Thr308) by everolimus and olaparib (Figure 4A). Phospho-MEK was also significantly decreased in all groups as compared to control. Western blot analysis of P-S6 and P-AKT confirmed RPPA results (Figure 4B).

In summary these results show that treatment by everolimus and olaparib, given as single agents and in combination, results in decreased expression of several proteins involved in DNA repair, chromatin remodeling and checkpoint control.

### The combination of mTOR and PARP inhibitors is also efficient in a basal-like BRCA2 breast cancer

Although BRCA2 mutations occur more frequently in ER+ breast tumors, several studies reported BRCA2 mutations in triple-negative breast cancer [16–18]. To test whether targeting mTOR and DNA repair could be a relevant therapeutic strategy in triple-negative breast cancer, we tested the combination of everolimus and olaparib in PDX model established from a BRCA2-mutated basal-like breast cancer [19]. This model carries a large deletion around the PTEN locus on chromosome 10q23 and shows loss of PTEN protein in IHC [20].

**Figure 5:**
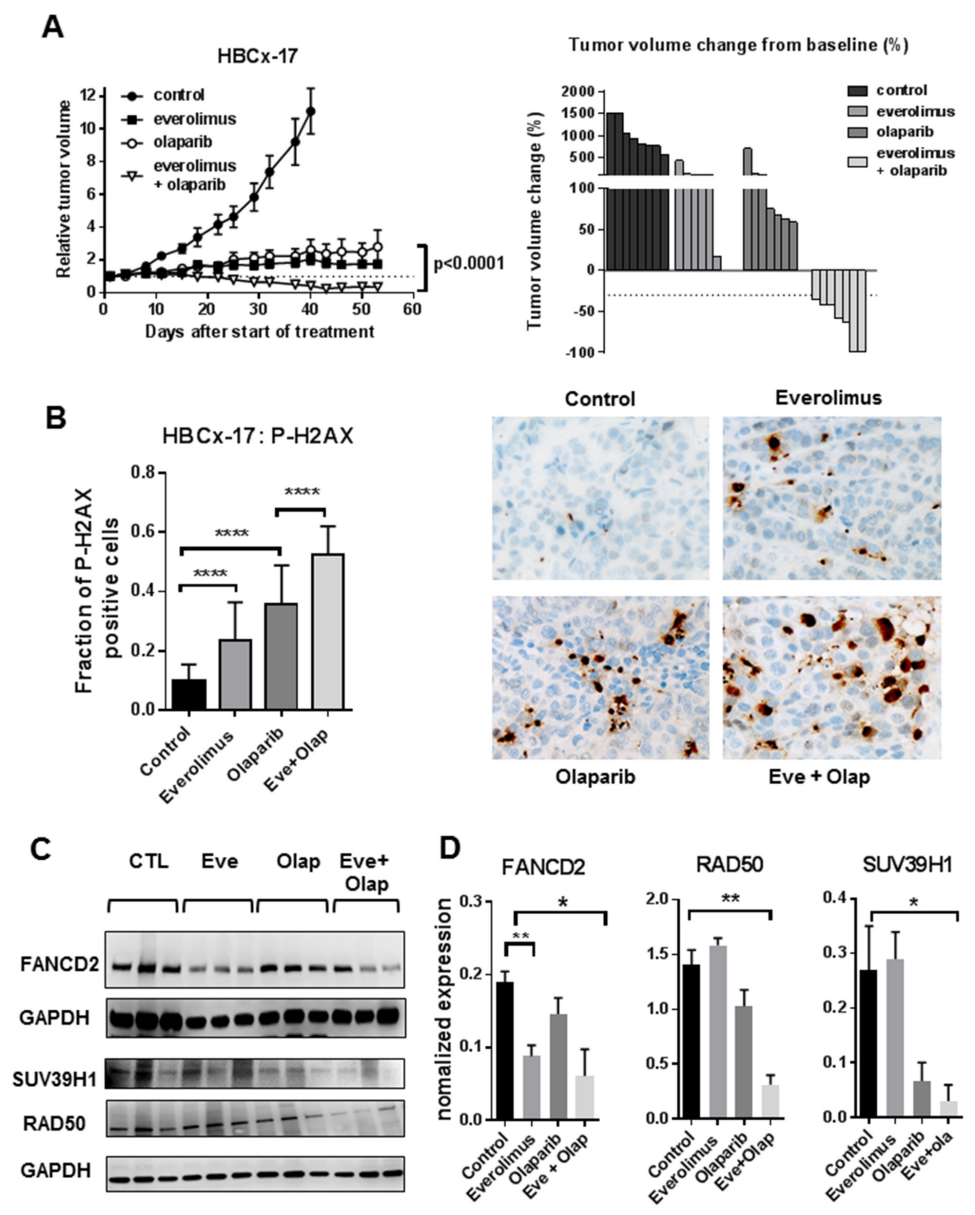
Anti-tumour activity of everolimus and olaparib in a BRCA2-mutated basal-like breast cancer **(A)** tumour growth curves of the HBCx-17 xenograft treated with olaparib, everolimus alone and in combination (left) and waterfall plot displayed as percent of tumour volume change from baseline (each bar is an individual xenograft**). (B)** Fraction of P-H2AX positive cells in HBCx-17 xenografts (N=5) and representative images of P-H2AX stainings (40X) **(C)** Western blot analysis of FANCD2, SUV38H1 and RAD50 expression. N=3. **(D)** Quantification of protein expression from western blot analysis.

Treatment by everolimus and olaparib in the monotherapy setting resulted in TGI of 80% and 78%, calculated at day 40 (when mice of the control group were sacrificed) (Figure 5A). Tumor growth inhibition were similar to those obtained in the HBCx22 TamR xenograft, however no tumor regressions were observed in the monotherapy setting. In the combination setting, tumor growth inhibition was of 96% at day 40 and at day 55, all mice showed tumor regression and 2 of them complete response (Figure 5A). To measure the extent of unrepaired DNA damage, we analyzed P-H2AX expression in formalin-fixed tumors harvested at the end of the experiment. DNA damage was increase in xenografts treated by everolimus and olaparib given in the monotherapy setting, with a marked increase in the combination arm where the fraction of P-H2AX positive cells was of 50% (Figure 5B). To confirm results found in the HBCx22-TamR model, we analyzed the expression of PI3K markers, DNA repair proteins (RAD50 and FANCD2) and SUV39H1 by western blot. Results, shown in Figure 5C and 5D, show a decreased expression of RAD50, FANCD2 and SUV39H1 expression in treated tumors. Inhibition of RAD50 was evident in tumors treated with the drug combination, while SUV39H1 was also inhibited in olaparib-treated tumors and FANCD2 in everolimus-treated xenografts. As for HBCx-22 TamR xenografts, formation of RAD51 foci was not detected in this xenograft, indicating homologous recombination deficiency (Supplementary Figure 1).

In summary, these results indicate a strong anti-tumor effect of the everolimus+olaparib combination in the BRCA2 basal-like breast cancer PDX HBCx-17, with evidence of increased DNA damage in all treatment arms and inhibition of key component of the homologous recombination pathway. Everolimus-induced inhibition of FANCD2 was confirmed in this PDX model.

### The combination of mTOR and PARP inhibitors is synergistic *in vitro* in a BRCA2 mutated cell line

To determine whether combination of olaparib and everolimus is synergistic in BRCA2 mutated cells, we performed drug combination cell viability assays in two triple-negative breast cancer cell lines, HCC1395 (BRCA2 mutated) [21, 22] and BT20 (PI3KCA mutated) [22]. HCC1395 cell line, but not BT20, has a BRCAness phenotype, as determined by the number of genomic large-scale transitions (LST) [23].

**Figure 6:**
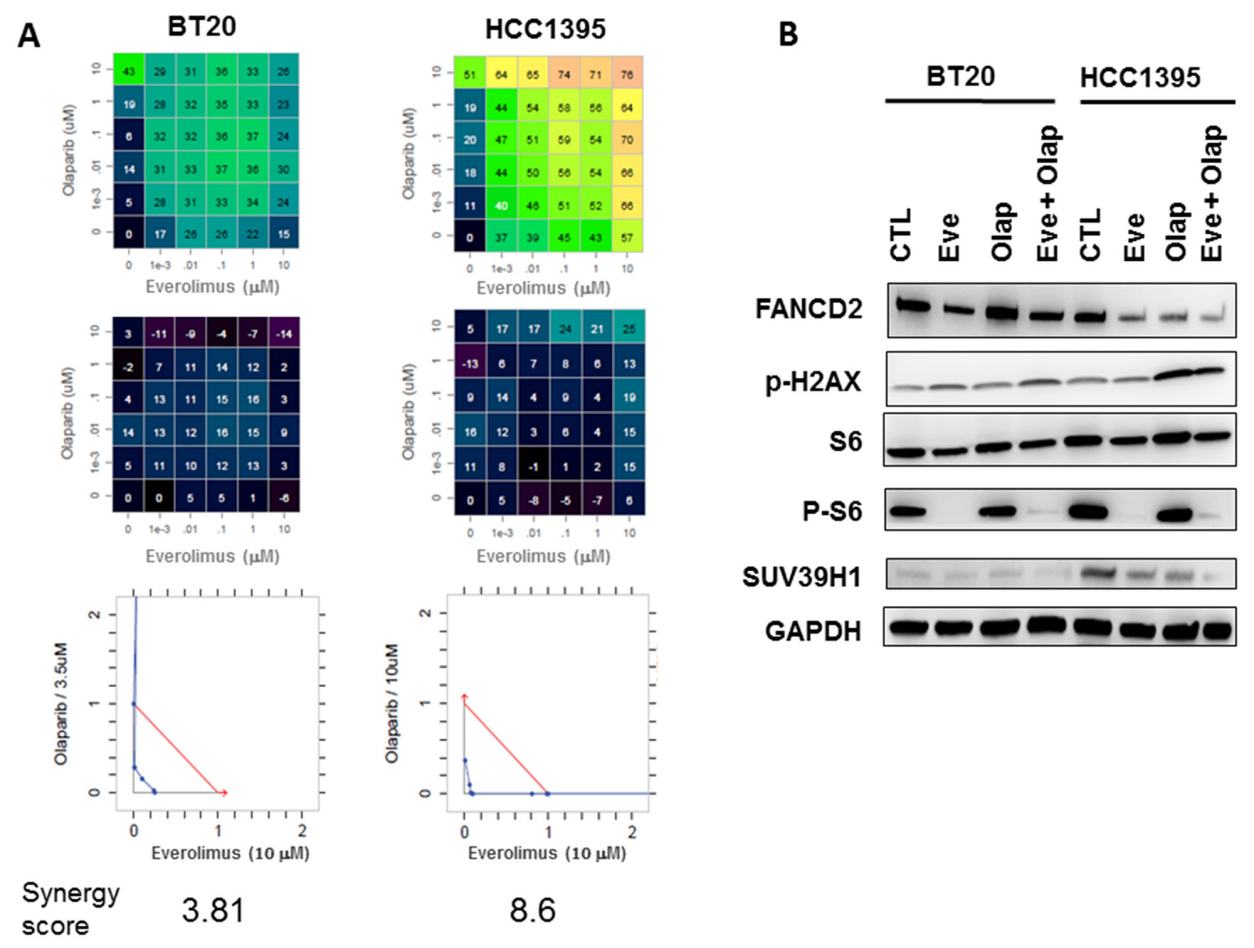
*In vitro* evaluation of everolimus and olaparib combinations in BT20 and HCC1395 breast cancer cell lines **(A)** Drug dose matrix data representing percentage of growth inhibitions (top panels), matrix with the loewe excess (middle panels) and isobolograms (bottom panels). **(B)** Western blot analysis of FANCD2, P-H2AX, S6/P-S6, SUV39H1 and GAPDH in BT20 and HCC1395 cell lines treated by everolimus and olaparib (10 μM).

Each combination was tested at multiple concentrations using a five-dose matrix. Olaparib and everolimus were tested from 0 to 10 μM. To quantify the combination strength, synergy scores and isobolograms were generated using the Loewe algorithm [24]. The matrix representing growth inhibition percentages and Loewe excess results are shown in Figure 6A. We observed that the combination of everolimus and olaparib were more synergistic in the HBC1395 cell line as compared to the BT20 cell line. The synergy scores were 8.6 and 3.81, respectively. Western blot analysis of cell lines showed inhibition of FANCD2 and SUV39H1 in the BRCA2-mutated HCC1395 cell line associated to increased expression of P-H2AX. P-S6 was equally inhibited by everolimus in BT20 and HCC1395.

## DISCUSSION

Hereditary breast cancer with germline mutations in *BRCA1* and *BRCA2* genes are characterized by a deficiency in DNA repair mechanisms that renders these tumors sensitive to platinum agents. In addition to platins, there has been increasing amount of information on the utility of the defects in DNA repair as targets for cancer therapy in BRCA-associated cancer [25]. Novel therapies like poly (ADP-ribose) polymerase (PARP) inhibitors are one of such example where the induction of double stranded breaks in DNA leads to cell death in tumor cell with homologous DNA repair deficiency [26].

So far combination treatment strategies have evaluated the efficacy and tolerability of olaparib with conventional chemotherapy, but recently several phase I trials have been launched to evaluate the tolerability of olaparib in combination with inhibitors targeting the PI3K/AKT/mTOR pathway. While PARP inhibitors and PI3K/AKT/mTOR inhibitors are effective when tested as single agents, cancers almost invariably develop resistance to these drugs through multiple mechanisms and find alternate ways to grow. This has prompted researchers to explore rational combinations of drugs that can help circumvent drug resistance. Breast cancers with germline BRCA mutations and activated PI3K pathway could be exceptional responder to a combination of DNA repair and PI3K pathway inhibitors.

In this study we tested the combination of two FDA-approved drugs, olaparib and everolimus, in PDX models established from BRCA2-mutated breast cancer carrying molecular alteration in the PI3K pathway. This association was first tested in the HBCx-22 TamR PDX, established from a ER+ breast cancer with a *PIK3R1* mutation and PTEN protein loss, and rendered resistant to tamoxifen *in vivo* [12, 13]. Remarkably, the drug combination given for 60 days resulted in 100 % of tumor regressions with 40% of complete responses, while in the monotherapy setting only 40% of animals showed tumor regression and no complete responses were observed. In a previous work, the everolimus efficacy was tested in this PDX combined to different endocrine therapies (tamoxifen, fulvestrant and estrogen deprivation) resulting at best in tumor stabilization [13]. Analysis of P-H2AX after treatment showed increased DNA damage not only in the olaparib-treated tumors, but also after everolimus treatment, suggesting a link between mTOR and DNA repair. To further explore the pathways and cross-talks potentially affected by everolimus and olarparib treatments, an RPPA analysis on several proteins involved in DNA repair, cell death and signaling pathways was performed on the HBCx22 TamR PDX treated with the different drugs. Interestingly, the expression of different DNA repair proteins involved in homologous recombination such as FANCD2, RAD50, P-P53BP1, was decreased by everolimus and strongly decreased in the combination arm, confirming that inhibiting mTOR is sufficient to affect DNA repair proteins. FANCD2 belongs to the Fanconi anemia pathway and is involved in the DNA damage response by cooperating with BRCA1/2 proteins in homologous-recombination (HR)-mediated repair [27]. Recently, it has been showed that BRCA-deficient tumors have a compensatory increase in FANCD2 activity that maintains fork stability and promote alternative end-joining DNA repair [28]. Loss of FANCD2 in BRCA1/2-deficient tumors enhances cell death revealing a synthetic lethal relationship between FANCD2 and BRCA1/2, and identifying FANCD2 as a central player orchestrating the choice of DNA repair pathway at the replication fork. The regulation of FANCD2 by mTOR has been also shown in rhabdomyosarcoma xenografts, where treatment by the dual mTOR inhibitor AZD8055 significantly enhanced radiosensitivity and melphalan through a downregulation of FANCD2 expression, suggesting that mTOR may promote the repair of DNA double-strand breaks by sustaining FANCD2 [29]. Additional evidence of the cross-talk between mTOR and FANC2 was found Guo et al., who showed that mTOR inhibitors sensitizes T-cell lymphoblastic leukemia for chemotherapy-induced DNA damage via suppressing FANCD2 expression [30]. The inhibition of FANCD2 in BRCA2-mutated xenografts might result in impaired end-joining and synthetic lethality in a context of homologous recombination deficiency. Additional experiments will be necessary to properly demonstrate impaired end-joining DNA repair in everolimus-treated tumors and to validate FANCD2 as a key component of the cross-talk between mTOR and DNA repair in breast cancer. In the HBCx22 TamR xenografts, we also found inhibition of SUV39H1 in everolimus-treated tumors and complete expression loss in tumors treated by everolimus and olaparib. SUV39H1 is a methyltransferase that is rapidly loaded into the chromatin at DSBs and methylates H3K9, resulting in ATM-dependent phosphorylation of DSB repair proteins [15]. It was recently shown that mTOR inhibitors impair homologous recombination repair and synergize with PARP inhibitors through a negative regulation of SUV39H1 in BRCA-proficient triple-negative breast cancer cell lines [31].

*In vitro* experiments performed in two breast cancer cell lines, one showing a BRCAness phenotype due to a BRCA2 mutation, show an increased synergy between olaparib and everolimus in the BRCA2 mutated cell line as compared to the BRCA wild-type (Figure 6). Inhibition of FANCD2 and SUV39H1 proteins were found only in the BRCA2-mutated cell line.

Other DNA repair proteins down-regulated in HBCx22 TamR by everolimus and olaparib treatments and strongly inhibited in the combination group, were RAD50, P-53BP1 and NBS1. Taken together these data suggest that inhibition of mTOR alone is sufficient to impair DNA repair and increase DNA damage, resulting in tumor regression and high percentage of complete response when combined to a PARP inhibitor.

The *in vivo* activity of olaparib has been also tested in combination with a PI3K inhibitor in PDX models of BRCA-proficient triple-negative breast cancer, where it resulted in increased tumor growth inhibition, but not in tumor regression, through a mechanism based on increased phosphorylation of ERK and BRCA expression down-regulation [32].

In our BRCA2 –mutated xenograft, we did not observe an increased phosphorylation of ERK nor a decreased in BRCA1 gene expression after mTOR inhibition, indicating that this mechanism is not involved in the anti-tumor effect of olaparib and everolimus combination.

The HBCx22 PDX model is the unique ER+ PDX of our cohort established from a BRCA2-mutated patient. As a consequence we could not validate our result in a second ER+ PDX model and tested the drug combination in a basal-like BRCA2 mutated PDX. BRCA2 mutated breast cancers are mostly luminal B (73%), while luminal A and basal-like breast cancer account for only 14% and 9%, respectively [33]. Although everolimus is approved only in ER+ breast cancer, it could be of potential interest to validate this drug combination in BRCA2 basal-like tumors. In the monotherapy setting, olaparib and everolimus treatment inhibited tumor growth, but no tumor regressions were observed. However, the association was as efficient as in the HBCx-22 TamR xenograft with 100% of animals showing tumor regression and 2 complete responses. Analysis of P-H2AX showed a marked increase of DNA damage in the combination setting as well as in both monotherapy arms and western blot analysis confirmed inhibition of FANCD2, RAD50 and SUV39H1 expression.

In conclusion, our results indicate that combining everolimus and olaparib in BRCA2 mutated breast cancer strongly inhibits expression of key proteins involved in DNA repair and results in massive DNA damage and tumor regression *in vivo*. The present findings suggest that the combination of mTOR and PARP inhibitors could represent a promising therapeutic approach for the treatment of BRCA2 mutated breast cancers. Additional experiments will be necessary to evaluate this combination strategy in other tumors such as BRCA1-mutated cancer and somatic breast cancer showing a BRCAness phenotype.

## MATERIALS AND METHODS

### PDX establishment and *in vivo* studies

Establishment of the PDX models HBCx-22 (ER+) and HBCx-17 (triple-negative) from primary BRCA2 mutated breast cancer was performed as previously described [12, 19]. Informed consent was obtained from patients before xenograft establishment. The HBCx22 TamR (tamoxifen-resistant) model has been established after tamoxifen treatment and tumor escape in an HBCx-22 xenograft [13].

Histology and IHC analysis of HBCx-17 and HBCx-22 PDX as compared to the original primary tumors have been previously published [12, 13, 19, 34, 35].

When tumors reached a volume of 60 to 200 mm3, mice were randomly assigned to the control or treated groups. Each group of treatment consisted of 7 or 8 mice. Everolimus and olaparib were purchased from Novartis and AstraZeneca, respectively. Everolimus and olaparib were administered orally at a dose of 15 mg/kg 3x/week and 100mg/kg 5x/week, respectively.

Experiments complied with the current laws of France and were approved by Institut Curie ethical committee.

Tumor volumes, tumor growth inhibition (TGI) and statistical significance of TGI were calculated as previously published [36, 37].

Percent change in tumor volume was calculated for each tumor using the following formula; [(Vf-V0)/V0]^*^100; where V0=Initial volume (at the beginning of treatment) and Vf= Final volume (at the end of treatment).

Classification of tumor response in waterfall plots: tumor regression, stabilization and progression corresponded to a percent of volume change lower, equal or greater than 0, respectively. Tumor sampling was performed 3 h after the last treatment. No specific toxicity was reported in the experiments.

### Breast cancer cell lines

The breast cancer cell lines BT20 and HCC1395 were purchased from the American Type Culture Collection cell lines (ATCC, LGC Promochem, Molsheim, France), authenticated by short tandem repeat profiling (data not shown) and cultured as previously described [38, 39].

### *In vitro* cell viability assay

*In vitro* cell viability assays were performed as described by Carita et al. [40]. Briefly, cells were seeded in three 96-well plates following a 6x6 matrix design. The day after, each drug was added following a matrix dilution format. 1:3 serial dilutions were tested to result in a total of six serial dilutions, including the DMSO control. Cell viability was measured after five days of drug treatment using the MTT assay (Sigma). Results were read using a spectrophotometer, and expressed as relative percentages of metabolically inactive cells compared with DMSO treated controls (percentage of growth inhibition). Combination effects were calculated with the Combination Analysis Module software, as previously described [24, 40]. A weighted “Synergy Score” was calculated across the dose matrix that adjusts for dose sampling and coverage and weights to favor combination effects at high inhibition levels.

### Immunohistochemistry

Xenografted tumors were fixed in 10% neutral buffered formalin, paraffin embedded, and hematoxylin–eosin (H&E) stained to differentiate the human tumor components from the murine stroma. Tumor tissues were analyzed by immunohistochemistry (IHC) for expression of P-H2AXSer139 (Mouse Monoclonal Antibody, clone JBW301, Merck Millipore, Billerica, MA) and Ki67 (rabbit monoclonal antibody, clone E18-E, Clinisciences, Nanterre – France).

Slides immunostained with mouse and rabbit IgG were used as negative controls. Slides were incubated with anti-rabbit/mouse secondary antibodies (horseradish peroxidase complex) and DAB (3,3′-diaminobenzidine tetrahydrochloride) as the substrate for color development (ChromoMap Kit with Anti rabbit OmniMap, Ventana Medical System).

P-H2AX scoring: homogenous nuclear staining and nuclei with 4 or more stained foci were considered positive for P-H2AX expression. For each tumor, the percentage of P-H2AX staining was evaluated in 7 different areas.

### Detection of RAD51 foci by immunofluorescence

Slides were incubated with a rabbit primary antibody anti-RAD51 (Ab-1, Merck Millipore, Billerica, MA). A HRP-conjugated goat anti-rabbit IgG (H+L) (1/500) and Alexa Fluor^®^ 594-conjugated Streptavidin (1/500) were used as the secondary antibodies. As positive control for RAD51 foci we used a BRCA wt breast cancer PDX that is resistant to cisplatin treatment and shows intrinsic high levels of RAD51 foci.

### Western blotting

Proteins were extracted from tumors using RIPA buffer (50 mM Tris HCL pH 8, 150 mM NaCl, 0.5% deoxycholic acid, 0.5% Triton), supplemented with protease and phosphatase inhibitors. Lysates were resolved on 10% agarose gels, transferred into nitrocellulose membranes (Bio-Rad, Hercules, CA, USA) and immunoblotted with rabbit antibodies against GAPDH, SUV39H1, FANCD2, RAD50, P-HIST. H3, AKT, P-AKT, S6 or P-S6 (Cell Signaling). After washes, membranes were incubated with the appropriate secondary antibodies horseradish peroxidase-conjugated affinity-purified goat anti–rabbit (Jackson ImmunoResearch Laboratories, Inc., Interchim). Quantification of P-AKT, FANCD2, RAD50 and SUV39H1 was performed by the Multi Gauge software and normalized on GAPDH expression.

### Reverse phase protein array (RPPA)

Samples were prepared as described in [41] and printed onto nitrocellulose covered slides (Supernova, Grace Biolabs) using a dedicated arrayer (2470 arrayer, Aushon Biosystems) in five serial dilutions (2000 to 125 μg/ml) and two replicates per dilution. Arrays were labeled with 64 specific antibodies (see Supplementary Table 1) as described in [41]. Read-out was done using IRDye 800CW (LiCOR) on an Innoscan 710-AL scanner (Innopsys). For staining of total protein, arrays were incubated 30 min in Super G blocking buffer (Grace Biolabs), rinsed in water, incubated 5 min in 0,000005% Fast green FCF (Sigma) and rinsed again in water. Raw data were normalized using Normacurve [42].

## SUPPLEMENTARY MATERIALS FIGURE AND TABLES







## Abbreviations

PDX: patient-derived xenografts
DSB: double-strand breaks
TGI: tumor growth inhibition
IHC: Immunohistochemistry
RPPA: Reverse Phase Protein Array
ER+: Estrogen receptor positive
TamR: tamoxifen-resistant
