# Supplementary material for: Inhibition of mTOR downregulates expression of DNA repair proteins and is highly efficient against BRCA2-mutated breast cancer in combination to PARP inhibition
Source: Oncotarget. 2018 Jul 3;9(51):29587–600. doi: 10.18632/oncotarget.25640 (PMC6049870; doi:10.18632/oncotarget.25640)
Supplement: Supplementary file 1 [file oncotarget-09-29587-s001.pdf]

Inhibition of mTOR downregulates expression of DNA repair proteins and is highly efficient against BRCA2-mutated breast cancer in combination to PARP inhibition

SUPPLEMENTARY MATERIALS

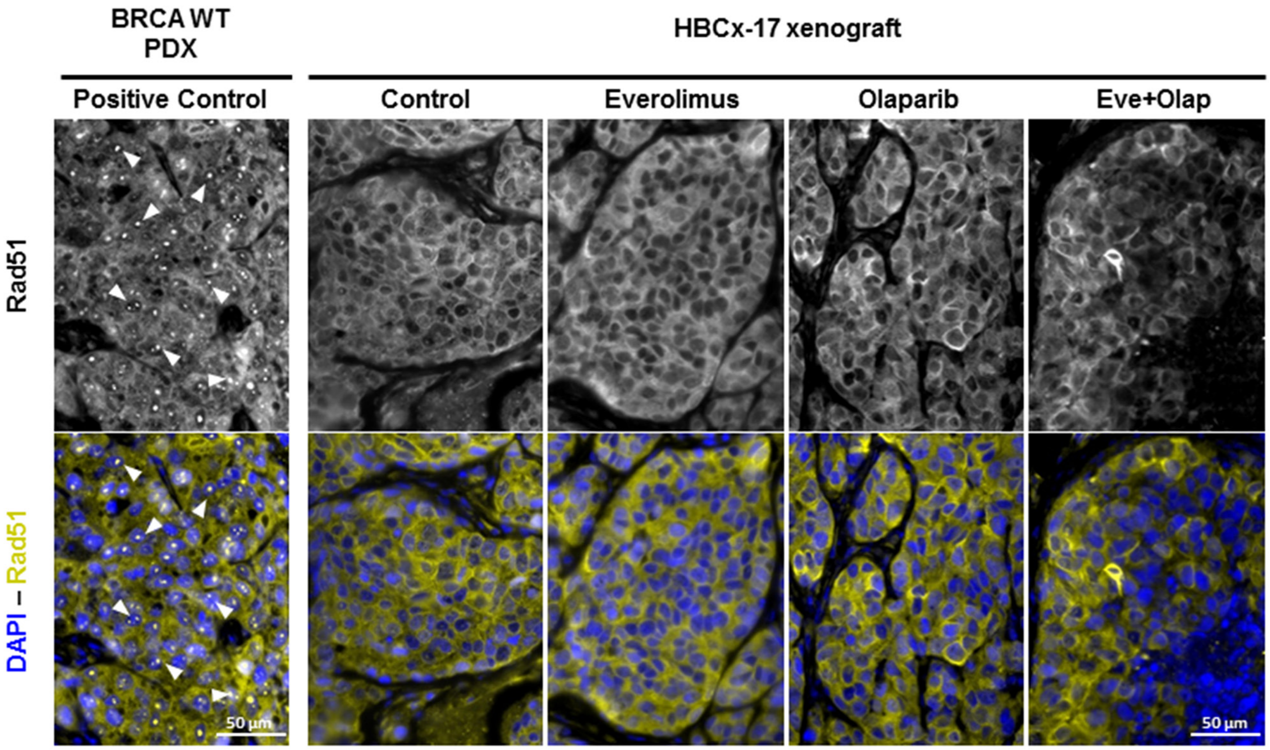

Supplementary Figure 1: Immunofluorescence analysis of RAD51 foci in the HBCx-17 xenograft.

Supplementary Table 1: List of antibodies tested in RPPA analysis.

See Supplementary File 1

Supplementary Table 2: Row data of RPPA analysis.

See Supplementary File 2
