# Supplementary material for: Inhibition of mTOR downregulates expression of DNA repair proteins and is highly efficient against BRCA2-mutated breast cancer in combination to PARP inhibition
Source: Oncotarget. 2018 Jul 3;9(51):29587–600. doi: 10.18632/oncotarget.25640 (PMC6049870; doi:10.18632/oncotarget.25640)
Supplement: Supplementary file 2 [file oncotarget-09-29587-s002.docx]

**Supplemetary Table S1: list of antibodies tested in RPPA analysis**

| **Name** | **Supplier** | **Reference** |
| --- | --- | --- |
| Phospho-Histone H2AX (ser139) | Abcam | 'ab2893' |
| Histone H2AX | CST | '2595' |
| Mre11(31H4) | CST | '4847' |
| Phospho-DNA-PK (Ser2612) | Epitomics | '2355-1' |
| Hsp90 alpha | Abcam | 'ab2928' |
| Phospho-Topoisomerase II a (Thr1343) | Epitomics | '1871-1 / ab52853' |
| ATM | Epitomics | '1549-1' |
| ERCC1 | CST | '3885' |
| Cleaved PARP (Asp214) p25 | Epitomics | '1051-1' |
| Phospho-ATM (ser1981) | Novus | 'NB110-55475' |
| MSH2 (D24B5) | CST | '2017' |
| 53BP1 | CST | '4937' |
| Rad50 | CST | '3427' |
| MDM2 [EP16627] | Abcam | 'ab178938' |
| Phospho-S6 Ribosomal Protein (Ser240/244) | CST | '2215' |
| YAP65 | Epitomics | '2060-1' |
| Phospho-S6 Ribosomal Protein (Ser235/236) | CST | '2211' |
| Akt (pan) (C67E7) | CST | '#4691' |
| Akt | CST | '9272' |
| Phospho-Akt (Thr308) (D25E6) | CST | '13038' |
| PTEN (D4.3) XP | CST | '9188S' |
| phospho-mTOR (Ser2448) | Abcam | 'ab109268' |
| Phospho-PTEN (ser380/Thr382/383) | CST | '9554' |
| Bcl2 | CST | '2876' |
| Bak | Epitomics | '1542-1' |
| mTOR | Abcam | 'ab51089' |
| Mst1/2 / STK3/4 | Bethyl | 'A300-468A' |
| Bcl-xL | Epitomics | '1018-1' |
| Mcl-1 | Santa-Cruz | 'SC-819' |
| Phospho-YAP65 (Ser127) | CST | '4911S' |
| PARP uncleaved p116 | Epitomics | '1077-1 / ab32378' |
| Bax (D2E11) | CST | '#5023' |
| Phospho-PDK1 (Ser241) | CST | '3061' |
| PDK1 (D37A7) | CST | '5662' |
| Phospho-Akt (Ser473) (193H12) | CST | '4058' |
| NBS1 p95 | CST | '3002' |
| Merlin | Epitomics | '3357-1' |
| Ape1 | CST | '4128' |
| p38 MAPK | Epitomics | '1544-1' |
| Topoisomerase II alpha | Epitomics | '1826-1' |
| Phospho-53BP1 (Ser1778) | CST | '2675' |
| Phospho-MEK1/2 (Ser217/221) | CST | '9154' |
| Phospho-Estrogen Receptor alpha (Ser118) | Epitomics | '1091-1' |
| p53 | CST | '9282' |
| Ezrin | Epitomics | '2255-1' |
| LATS1 | Bethyl | 'A300-478A' |
| MEK1/2 | CST | '9122S' |
| Phospho-Chk2 (Thr68) | CST | '2197' |
| Phospho-p38 MAPK (Thr180/Tyr182) | CST | '4631' |
| Phospho-p44/42 MAPK (Thr202/Tyr204) | CST | '4377 (197G2)' |
| Estrogen receptor alpha (D8H8) | CST | '8644' |
| p44/42 MAPK | CST | '9102' |
| Phospho-Chk1 (Ser280) | CST | '2347' |
| Phospho-Ezrin (Thr567) /Radixin (Thr564)/Moesin (Thr558) | CST | '3141' |
| Phospho-FANCD2 (Ser222) | CST | '4945' |
| S6 Ribosomal Protein (5G10) | CST | '2217' |
| FANCD2 | Epitomics | '2986-1' |
| Histone H3 trimethylated K9 (H3K9me3) | Upstate Millipore) | '07-442' |
| Phospho-p53 (Ser15) | CST | '9284' |
| SUV39H1 | CST | '8729' |
| Rad51 (D4B10) | CST | '8875' |
| Progesterone receptor | Epitomics | '1483-1' |
| Ku80 (C48E7) | CST | '2180' |
| Phospho-Progesterone Receptor (Ser190) | Epitomics | '2258-1' |
